# Discovery of an unidentified species of nicothoid copepod infesting cancrid crabs in Santa Barbara, California

**DOI:** 10.1002/ecy.70263

**Published:** 2025-12-09

**Authors:** Jaden E. Orli, Sophia M. Lecuona, Gabrielle O. Plewe, Carson N. Gadler, Armand M. Kuris, Danny Tang, Zoe L. Zilz

**Affiliations:** ^1^ Ecology, Evolution, and Marine Biology, and Marine Science Institute University of California Santa Barbara Santa Barbara California USA; ^2^ Bren School of the Environment, University of California Santa Barbara Santa Barbara California USA; ^3^ School of Dentistry, University of California San Francisco San Francisco California USA; ^4^ Environmental Services Department Orange County Sanitation District Fountain Valley California USA

**Keywords:** *Choniosphaera*, copepod, decapod, egg predator, nicothoid, rock crab

Externally brooding crustaceans host a variety of symbiotic egg predators, often causing substantial brood mortality (Kuris et al., 1991; Kuris & Wickham, 1987). Taxa known to live and feed on crustacean eggs (summarized by Kuris, 1993) include various microorganisms, rotifers, gastropods, worms (nemerteans, turbellarians, nematodes, polychaetes, and oligochaetes), and other small crustaceans (copepods, amphipods, and isopods). Heavy infestations of egg predators, for example, nemertean worms in the Dungeness crab, *Metacarcinus magister*, have been implicated in the collapse (Wickham, 1979, 1986) and slow recovery (Hobbs & Botsford, 1989; Kuris et al., 1991) of some crustacean fisheries. The copepod family Nicothoidae includes parasites and symbiotic egg predators of other crustaceans, most of which have adopted egg mimicry as a life history strategy (Boxshall & Halsey, 2004). High intensities of nicothoids have negatively impacted the fecundity of commercially important species, such as the blue sand crab in Australia (Shields & Wood, 1993).

In October 2021, during a routine classroom laboratory activity at the University of California Santa Barbara focused on the demonstration of crustacean egg predators, we observed, for the first time, a nicothoid copepod in the genus *Choniosphaera* Connolly, 1929 infesting ovigerous yellow rock crabs (*Metacarcinus anthonyi* Rathbun, 1897). This discovery prompted further investigation, during which crabs were collected by fishermen from a 66–100 m depth using baited crab pots along the Gaviota Coast, west of Santa Barbara, CA (see Appendix S1: Section S1 for full methods). Crabs were held in flow‐through aquaria during the investigation. We discovered *Choniosphaera* sp. in abundance on two other commercially important rock crab species, *Romaleon antennarium* Stimpson, 1856 and *Cancer productus* Randall, 1840. These crabs are habitat generalists, occurring in coastal waters from 0 to 150 m, and together form a small fishery in California (CDFW, 2019; Morris et al., 1980). Four species of nicothoids have been reported in association with seven species of brachyuran crabs, with varying effects on host fecundity (Bloch & Gallien, 1933; Connolly, 1929; Dang et al., 2022; Fischer,  1956; Gnanamuthu, 1954; Johnson, 1957; Santos & Björnberg, 2004; Shields & Wood, 1993). Nicothoid copepods have not been previously reported infesting decapod species from the Eastern Pacific (Appendix S1: Figure S1) despite extensive inspection of cancrid egg clutches during studies of the egg‐predatory nemertean *Carcinonemertes epialti* Coe, 1902 in the 1990s (Shields et al., 1990, 1991). Furthermore, the University of California Santa Barbara Invertebrate Biology undergraduate class and instructors have carefully inspected cancrid egg masses for symbiotic egg predators annually since 1978. This study documents the first detection of a nicothoid copepod in these examinations. Members of two nicothoid genera, *Choniosphaera* and *Carcinothoe*, are reported to infest brachyuran crab egg masses (Bloch & Gallien, 1933; Connolly, 1929; Fischer, 1956; Lee & Kim, 2024). The nicothoid discussed herein is more closely aligned with *Choniosphaera* based on adult female morphological characteristics, including a globular shape with no abdomen, distinctly ventrally positioned mouthparts, a biarticulate exopod and uniarticulate endopod on legs 1 and 2, antennules with 11 segments, and a caudal ramus with four to five setae (Bloch & Gallien, 1933; Connolly, 1929; Lee & Kim, 2024).

The adult female nicothoid was the first life stage we observed on crab eggs (Figure 1a). We did not observe any adult nicothoids that we could recognize as male, and the apparent lack of male *Choniosphaera* sp. warrants further investigation. Adult females of *Choniosphaera* sp. appear to live permanently on the egg masses of their crab hosts and as such, their morphology is highly modified. The adult female body is globular and not visibly segmented. Its shape is similar to the host's eggs, and it camouflages well with the egg mass (Figure 1b; Appendix S1: Figure S3). Adult female *Choniosphaera* sp. change coloration as they increase in size and become gravid (Figure 1, see also Adult_Nicothoid_Video.MOV in Orli et al., 2025b). Adult nicothoids found on host egg masses that are in early developmental stages range in color from yellow to orange, while adults found on later stage egg masses are maroon (Figure 1a, see Appendix S1: Table S1 for a description of host egg developmental stages). We observed that adult female *Choniosphaera* sp. increase in size without molting, which is a phenomenon that has been documented for other symbiotic copepod females (Kabata, 1979; Ohtsuka et al., 2004, 2005; Smith & Whitfield, 1988). When adult females of *Choniosphaera* sp. were removed from the host eggs, all died within 24 h despite being kept well aerated in flow‐through seawater and provided with eggs to feed on.

**FIGURE 1:**
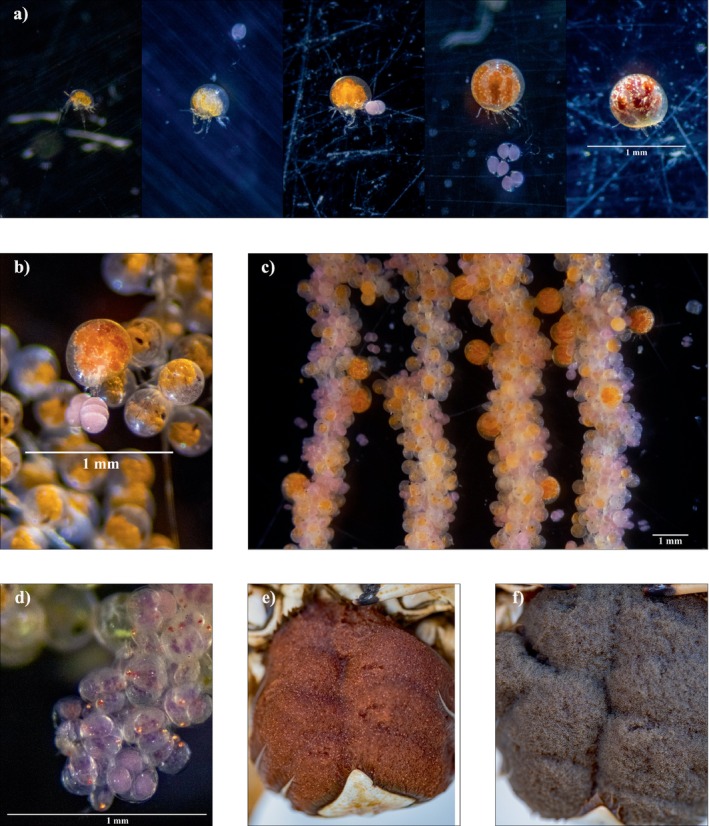
Adult nicothoids from *Metacarcinus anthonyi*; (a) sequence of sizes and colors of adult nicothoids; (b) adult nicothoids and their eggs (pink) among crab egg fascicles and several empty crab eggs whose embryos were consumed by nicothoids are also visible (viable crab eggs are distinguished by orange color and eyespots); (c) gravid adult nicothoid with egg sacs; (d) nicothoid egg sacs containing up to four embryos in each sac, note their red eyespots; (e) heavily infested crab egg mass, appearing maroon due to the great number of pink nicothoid eggs; (f) crab egg mass at the same developmental stage as (e), but with a comparatively low infestation of nicothoids. Photo credits: J. E. Orli.

The *Choniosphaera* sp. life cycle appears to be coupled with host crab egg development (Appendix S1: Figure S2). Throughout the duration of host egg development (approximately 45 days for *M. anthonyi*; see Appendix S1: Table S1), adult females of *Choniosphaera* sp. oviposit egg sacs, carrying between one and four egg sacs at a time. As the females move throughout the host egg mass, their egg sacs become tangled among the host's eggs (Figure 1d). Each sac contains one to four embryos (Figures 1b and 2a). We were unable to track the number of eggs deposited per female throughout host egg development because it was impossible to distinguish individual nicothoids in situ, and the tendency of females to die when removed from the host's egg mass prohibited prolonged observation ex situ. The nicothoid copepod examined in this study follows a life cycle similar to the other two *Choniosphaera* species, *Choniosphaera cancrorum* and *Chonios. maendis* (Bloch & Gallien, 1933; Connolly, 1929). Of the 22 genera in the family Nicothoidae, only two genera, *Choniosphaera* and *Choniomyzon*, contain species where eggs hatch into nauplii (Wakabayashi et al., 2013). Once nauplii hatch from the egg sac (Figure 2b), they develop more appendages and increased segmentation through an unknown number of sequential molts until they reach the first copepodid stage, which is characterized by well‐developed swimming legs (Figure 2d). The density of copepodids peaks near the end of host egg development (Appendix S1: Figure S2). Copepodids were observed feeding on host eggs.

**FIGURE 2:**
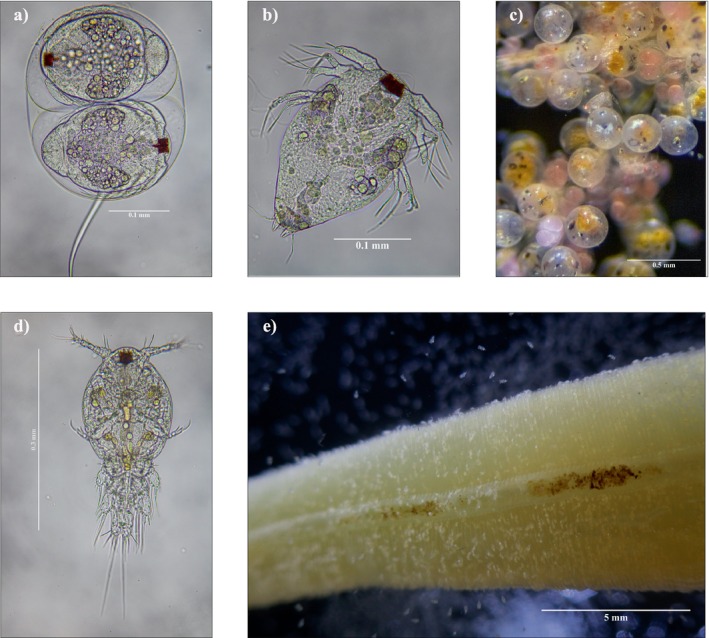
Larval nicothoid copepods taken from *Metacarcinus anthonyi* egg masses and gills; (a) two embryos within an egg sac, about to hatch into nauplii within an egg sac; (b) nauplius larva; (c) various nicothoid copepodids in situ in host egg mass. Note the red eye spots; (d) copepodid larva; (e) gill excised from *M. anthonyi* infested with nicothoid copepodids. Photo credit: J. E. Orli. (a–d) and G. O. Plewe (e).

Once the crab zoeae hatch from the host crab egg mass, the female crab removes old egg shells, aborted eggs, and debris from her pleopods (as noted by Shields et al., 1991), presumably including all nicothoids remaining on the egg mass. However, we also observed copepodids among host crab gill lamellae both before and after host egg hatching (Figure 2e). Other symbiotic egg predators of crabs (Kuris, 1993) have been reported from host gills, notably including a nicothoid, *Carcinothoe indica*, that is closely related to the species described herein (reported as *Choniosphaera indica* in Shields & Wood, 1993). We detected nicothoids in the egg masses of seven crabs that oviposited a subsequent clutch of eggs while under observation. Copepodid larvae were observed in these crabs' egg masses within a day of oviposition, and adult *Choniosphaera* sp. were present within 9 days (Appendix S1: Figure S2). Based on these observations, we speculate that copepodids migrate to the gills to wait for their host's next oviposition, but we did not observe copepodids moving from the host egg mass to the host gills. In at least one other nicothoid species, subadult females have been observed migrating through the host body (Ohtsuka et al., 2007). We also recovered copepodids in the gills of male host crabs, suggesting that the copepodid stage may be able to disperse to new hosts and that male crabs may serve as reservoir hosts (Appendix S1: Table S2). The nauplii could also potentially disperse, but because of their lack of developed swimming legs, they would likely require physical contact between hosts to do so (Figure 2b).

We observed *Choniosphaera* sp. in *R. antennarium*, *M. anthonyi*, and *Can. productus* egg masses from Santa Barbara, CA, and San Diego, CA. Nearly all crabs of both sexes, for all three species examined, had at least one life stage of nicothoid in their gills, their egg masses, or both (Appendix S1: Table S2). All ovigerous *M. anthonyi* and the majority of the other two ovigerous cancrids examined had reproductive nicothoid adults present in the egg mass. The only crabs uninfested by any nicothoid life stages (including the gills) were two juveniles, a non‐ovigerous female, and two males that had been housed for over 6 months (Appendix S1: Table S2). It is possible that copepodids cannot survive in the gills indefinitely and that they must feed on egg yolk in order to reach adulthood, which would explain the observed loss of infection in male crabs. The lack of nicothoids in juvenile crabs leads us to hypothesize that nicothoids do not colonize potential hosts until the crabs are sexually mature.

It is noteworthy that a distinctive nicothoid egg predator suddenly appeared in abundance on cancrid crabs in the Santa Barbara area. Nicothoid copepods had never been observed despite their distinctive appearance. This discovery suggests two mutually exclusive explanations: either this is an exotic introduction, or it is a native species which had previously existed unobserved on other crabs and has now transferred to a new group of hosts. The exotic hypothesis is unlikely because no congeneric nicothoid is known from the Indo‐Pacific region, temperate, or boreal Pacific waters. A species in the similar genus *Choniomyzon* (D. Tang, personal communication) is common on *Portunus pelagicus* from India to Australia. However, historical studies on the two Atlantic species of *Choniosphaera* best support the hypothesis that nicothoids are native but perhaps ephemeral, emerging in abundance episodically over a time scale of decades. *Choniosphaera maenadis* was discovered on *Carcinus maenas*, the common and well‐studied European shore crab, at Wimereux, Normandy in the mid‐1930s (Bloch & Gallien, 1933; Gallien & Bloch, 1936), and reported from Whitstable, England, about 100 km from Wimereux by Gordon (Needham, 1933). It was not reported again until the 1950s from the North Sea, Germany (Fischer, 1956), and Norfolk, England in 1959 (Hamond, 1973). In both instances, *Chonios. maenadis* appears to have become locally abundant only to never be reported again (see also Stentiford, 2008). Similarly, in New England and the Canadian Maritimes, *Chonios. cancrorum* has been reported only twice, many years apart (Connolly, 1929; Johnson, 1957).

The presence of *Choniosphaera* sp. represents a threat to the under‐managed and data‐poor California rock crab fishery (Fitzgerald et al., 2018). An egg predator that is present in 100% of the breeding host population will likely impact population fecundity (see Appendix S1: Section S2 for further discussion). At the time that *Chonios. cancrorum* was discovered in the brood of *Carcinus maenas* in New England, the effect of *Chonios. cancrorum* was notable enough to garner hope for its use as a biological control of that invasive pest species (Johnson, 1957). The discovery of this novel egg predator in Southern California could have a deleterious effect on the Santa Barbara rock crab fishery and the general role of these cancrids as predators in nearshore subtidal habitats. Notably, these nicothoids add to brood losses caused by *Carcinonemertes* sp. nemertean egg predators (Shields et al., 1990). The sudden emergence of an epizootic of nicothoid egg predators combined with a robust population of existing egg predators warrants further research on their impact on the fecundity of host populations. Our research team is currently quantifying egg mortality rates in infested egg masses. Further research should focus on revealing the current geographical extent of the nicothoid outbreak and the diversity of its host range among Eastern Pacific crabs.

## CONFLICT OF INTEREST STATEMENT

The authors declare no conflicts of interest.

## Supporting information


Appendix S1.

## Data Availability

Data and code (Orli et al., 2025a) are available in Dryad at https://doi.org/10.5061/dryad.rv15dv4fw. Video files (Orli et al., 2025b) are available in Zenodo at https://doi.org/10.5281/zenodo.10699477.
