# Supplementary material for: Discovery of an unidentified species of nicothoid copepod infesting cancrid crabs in Santa Barbara, California
Source: Ecology. 2025 Dec 9;106(12):e70263. doi: 10.1002/ecy.70263 (PMC12687570; doi:10.1002/ecy.70263)
Supplement: Supplementary file 1 — Appendix S1. [file ECY-106-e70263-s001.pdf]

Appendix S1

Journal: Ecology

Manuscript Title: Discovery of an unidentified species of nicothoid copepod infesting cancrid crabs in Santa Barbara, California

Authors: Jaden E. Orli, Sophia M. Lecuona, Gabrielle O. Plewe, Carson N. Gadler, Armand M. Kuris, Danny Tang, Zoe L. Zilz

Section S1. METHODS

S1.1. Crab Collection and Housing

After the initial observation of nicothoid egg predators in *Metacarcinus anthonyi* egg masses in 2021, we obtained rock crabs from baited crab pots set by local fishermen along the Gaviota Coast of Santa Barbara County to determine the prevalence and abundance of the nicothoid. We collected data from 65 ovigerous females (54 *M. anthonyi,* 4 *Romaleon antennarium,* and 7 *Cancer productus*), 23 males (12 *M. anthonyi,* 7 *R. antennarium,* and 4 *C. productus*), and 1 juvenile female *C. productus*. Crabs were housed in individual tanks with an independent source of running filtered seawater. Upon collection we recorded carapace width to 1 mm, abdomen width to 1 mm, weight the nearest gram, injuries, and initial egg development stage or EDS (Appendix S1: Table S1).

S1.2. Host Crab Brood Examination

All ovigerous female crab egg masses (n = 65) were routinely examined for the presence of nicothoid copepods. Every three days we collected a sample of at least one thousand crab eggs from the center of a haphazardly selected pleopod by removing a few fascicles of setae with attached eggs. The total number of crab eggs per sample was counted and empty eggshells and eggs with evidence of arrested development were quantified. This process was repeated for each ovigerous female until the crab hatched her brood. Most post-ovigerous crabs were released but

some were dissected to inspect the gills and other internal organs for nicothoids (see Host Crab Dissections). Halfway through egg development (EDS E and later; Appendix S1: Table S1) we also quantified nicothoid egg sacs and larval/juvenile stages. We calculated the density of nicothoids per crab egg and then averaged per EDS. Because *Metacarcinus anthonyi* fecundity ranges from 700,000 to 3,300,000 eggs per brood (Shields et al., 1990) we extrapolated density to calculate abundance of nicothoids per 1,000,000 eggs to estimate total nicothoid abundance per crab (Appendix S1: Figure S2). To observe movement and feeding behavior of the nicothoids, we placed a single adult nicothoid with a fascicle of 100 to 200 host eggs in a mesh container with flowing seawater until that nicothoid died, which usually occurred within a few hours.

S1.3. Host Crab Dissections

To determine the presence of the nicothoid in other Southern California crab species, we dissected 28 *M. anthonyi,* 8 *R. antennarium,* and 7 *C. productus.* Crabs were humanely euthanized by pithing through the ventral thorax directly over the thoracic ganglion. We removed the two center gills from both the left and right side of the crab and placed them in petri dishes with seawater. Each gill was examined under a dissecting microscope for nicothoids, damage or scarring, and the accumulation of a microbial biofilm. We randomly selected one gill, counted the lamellae, and then rinsed the gills with ethanol to immobilize any attached nicothoids which allowed us to count them. We recorded the number and life stage of nicothoids per gill lamellae. Samples of other organs, including gonads and digestive tissue, were also examined for the presence of nicothoids.

Section S2: POTENTIAL IMPACTS ON HOST REPRODUCTION

Based on our observations of nicothoid feeding activity

(Adult_Nicothoid_Feeding_Video.mov in Orli et al. 2025) and its impact on host eggs (Main Text: Figure 1b) we hypothesize that nicothoids can have a substantial effect on the fecundity of individual crabs. Although feeding rates of adults or copepodids have not yet been quantified, we document high infestations of nicothoid adults resulting in near total loss of host eggs on some ovigerous hosts. Nicothoids become so numerous that they can effectively replace host eggs with their own eggs (Infested_Host_Eggs_Video.mov in Orli et al. 2025). Copepodid presence in the gills of non-gravid crabs and the prompt appearance of copepodids in newly oviposited egg masses suggests that copepodids migrate to the gills in between broods, returning to the host eggs upon subsequent oviposition. This behavior allows for a female crab to be infested for the entirety of her reproductive period. The larval nicothoids present in the gill chamber presumably function as the baseline population for infestations in future, subsequent, broods (Appendix S1: Table S1). Samples from subsequent broods showed higher abundances of adult nicothoids and nicothoid eggs than on prior broods. We examined crabs immediately post-molt and recovered copepodids from the gills, indicating that the crabs retain infestations following a molt, but the mechanism of this retention is unknown. Uninfested female crabs may initially become infested upon contact with an infested male crab. Coupled with the continuous reproduction of *M. anthonyi* and *R. antennarium* throughout the year (Shields et al. 1990), continuous infestation from the gill sites likely contributes to the proliferation of this novel nicothoid epizootic (Highly_Infested_Host_Gills_Video.mov in Orli et al. 2025). No negative

impact on the host crab has been observed as a result of the presence of the copepodids in the host gill chambers, but highly infested gills, like those we observed, potentially result in reduced gas exchange (Main Text: Figure 2e, Appendix S1: Table S1).

TABLE S1. *Metacarcinus anthonyi* crab egg development stages (EDS). Illustrations by Z. L. Zilz and photographs by J. E. Orli.

Stage	Description	Average Duration (days)	Sketch	Image
A	Between 1 and 64 cells, all yolk. 10% developed.	4.1 days	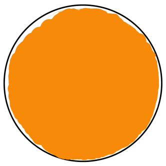	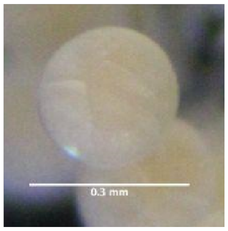
B	One small clear spot visible.	3.7 days	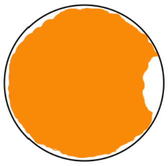	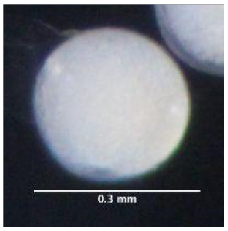
C	2 small clear spots visible.	1.7 days	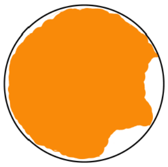	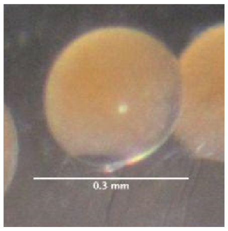
D	One large spot is visible, but no segmentation visible yet. About 20% developed.	2.6 days	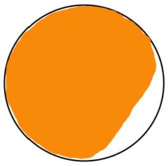	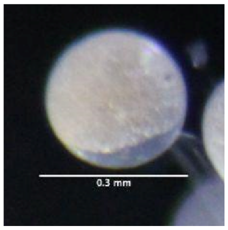
E	Segmented appendages (tagmata) visible below large clear spot. About 40% developed.	11.9 days	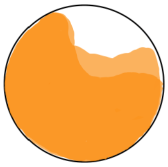	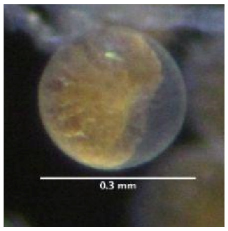

**F**	Eyespots visible for the first time. 55% developed.	2.0 days	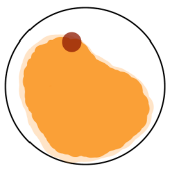	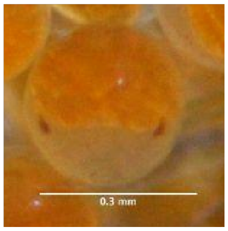
**G**	Abdominal pigment visible. 60% developed.	2.3 days	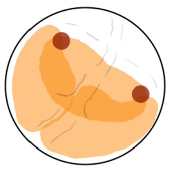	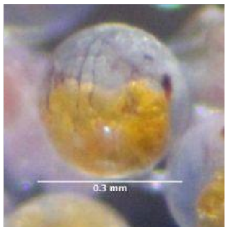
**H**	Yolk has started to divide into lobes, heartbeat is visible. 67% developed.	1.7 days	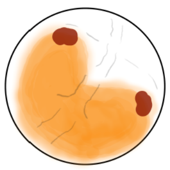	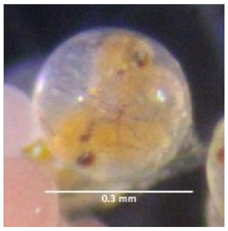
**I**	Yolk is 4 lobed. 75% developed.	6.9 days	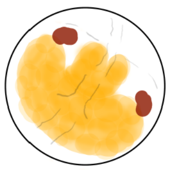	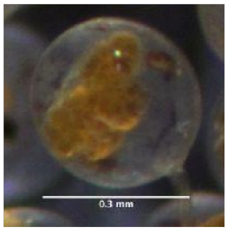
**J**	Yolk is two-lobed but lobes are still connected. 85% developed.	2.0 days	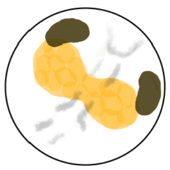	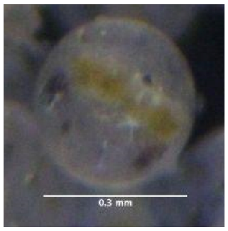
**K**	Yolk is two lobed and separated. 90% developed.	2.1 days	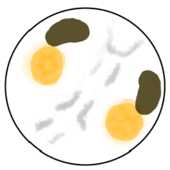	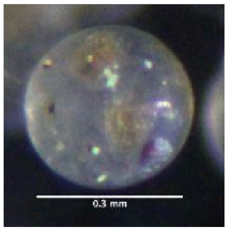

**L**	Yolk absent, lots of “flexure” i.e. movement within the egg casing.	1.5 days	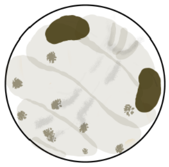	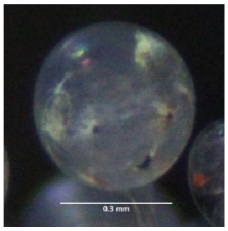
**M**	Active hatching.	0.8 days		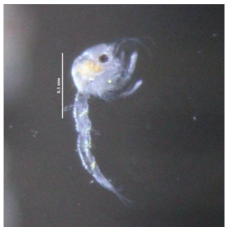

TABLE S2. Summary of prevalence and intensity of feeding nicothoid stages (copepodids and adults) in male and female crab hosts, by species.

Species	Sex	Prevalence Infested	Prevalence with Adults in Host Egg Mass	Mean Intensity of Copepodids in Host Gill Chamber
Metacarcinus anthonyi	F	100% (n = 55)	100% (n = 54)	14,282 ± 21,755 (n = 19)
	M	83% (n = 12)	NA	235 ± 373 (n =10)
Cancer productus	F	66% (n = 9)	100% (n = 4)	24 ± 31 (n = 3)
	M	100% (n = 4)	NA	42 ± 59 (n = 4)
Romaleon antennarium	F	66% (n = 6)	100% (n = 2)	4,608 ± 5,578 (n = 4)
	M	100% (n = 8)	NA	61 ± 34 (n = 7)

Notes: Figures are means ± standard deviation. Individuals were considered infested if any nicothoid life stage (adult, copepodid, nauplius, or egg sac) was confirmed to be present during any evaluation. The intensity of copepodids in the host gill chamber was scaled to a total of 12 host gills.

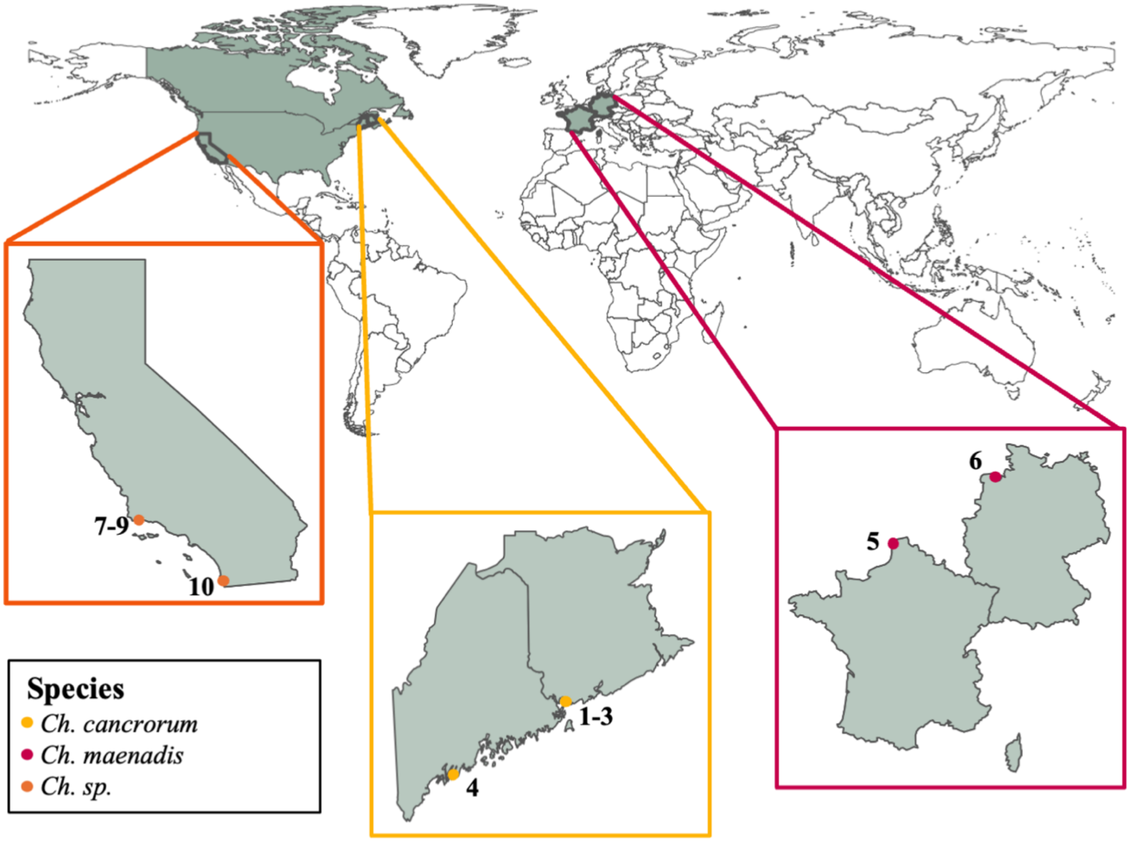

FIGURE S1. Global distribution of species in the genus *Choniosphaera*, the most likely parent genus of the nicothoid reported in this publication. Individuals of this genus have previously only been identified in the Atlantic Ocean. Numbers indicated on the map correspond to the first documentation of each *Choniosphaera* species infesting a novel host species at that location. Individuals of this genus were found infesting (1) *Cancer irroratus*, (2) *Atelecyclus undecimdentatus*, and (3) *Cancer borealis* in Passamaquoddy Bay (Connolly, 1929), (4) *Carcinus maenas* in Linekin Bay (Johnson, 1957), (5) *C. maenas* in the English Channel (Bloch and Gallien, 1933), (6) *C. maenas* in the Wadden Sea (Fischer, 1956), in the Pacific Ocean; (7) *Metacarcinus anthonyi*, (8) *Cancer productus*, and (9) *Romaleon antennarium* on the Gaviota Coast (this publication), and (10) *M. anthonyi* in San Diego (this publication).

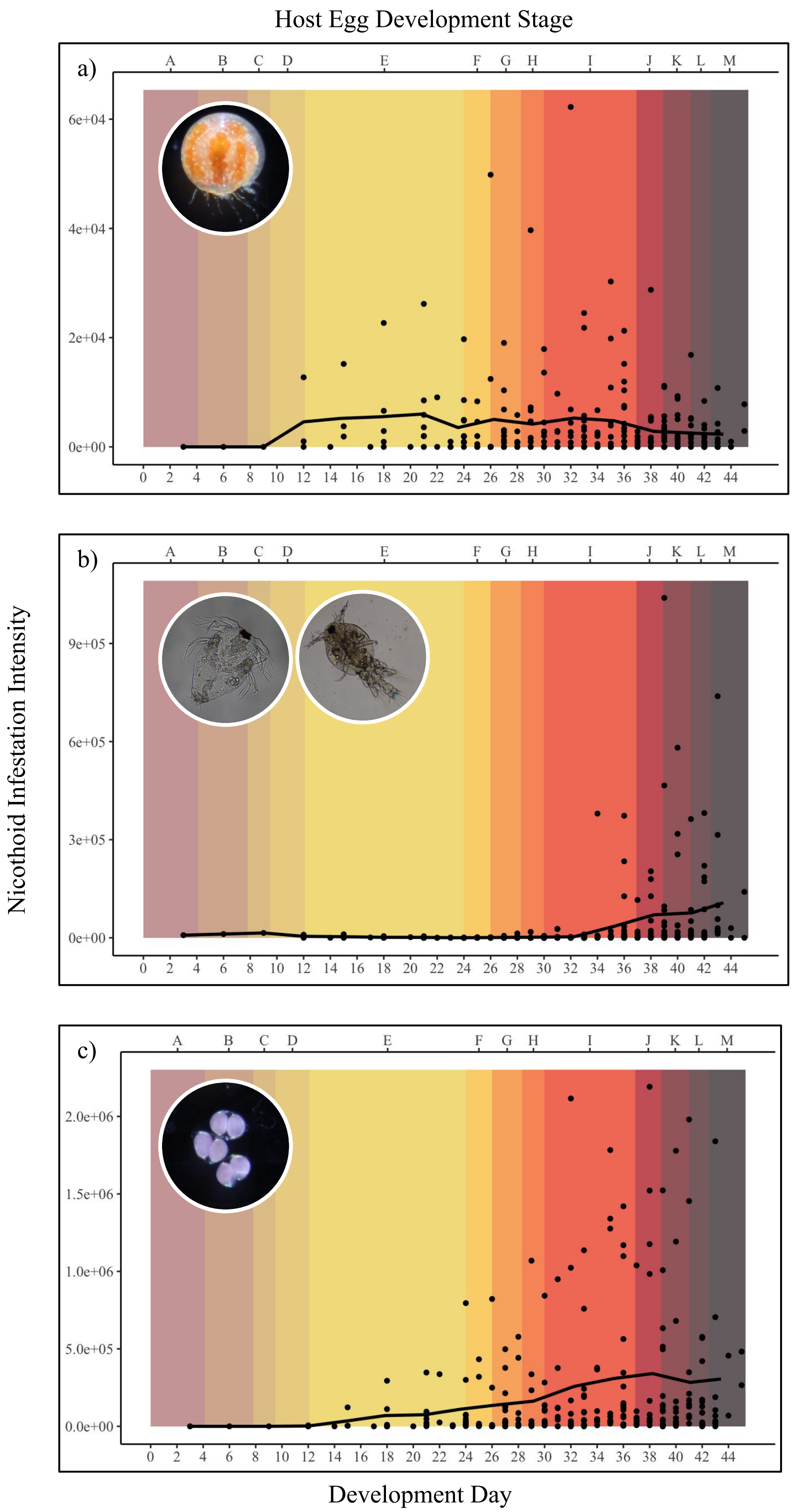

FIGURE S2. Estimated nicothoid intensity throughout the duration of host crab brooding period for (a) adult nicothoids (n = 41 crabs), (b) larval nicothoids (n = 41 crabs), (c) nicothoid eggs (n = 41 crabs). Points represent estimated nicothoid intensities for an individual crab on a particular "development day" (day since host-egg deposition). The solid lines represent the mean estimated intensity of nicothoids per crab egg clutch. Nicothoid intensity was estimated by calculating the density of nicothoids per host egg, averaging every three days, and then scaling to one million host eggs to approximate intensity in an entire host brood. Colored bars correspond to the host egg development stages outlined in Table S1. Some individual crabs were only examined for a portion of their brooding duration. Photo credits J. E. Orli.

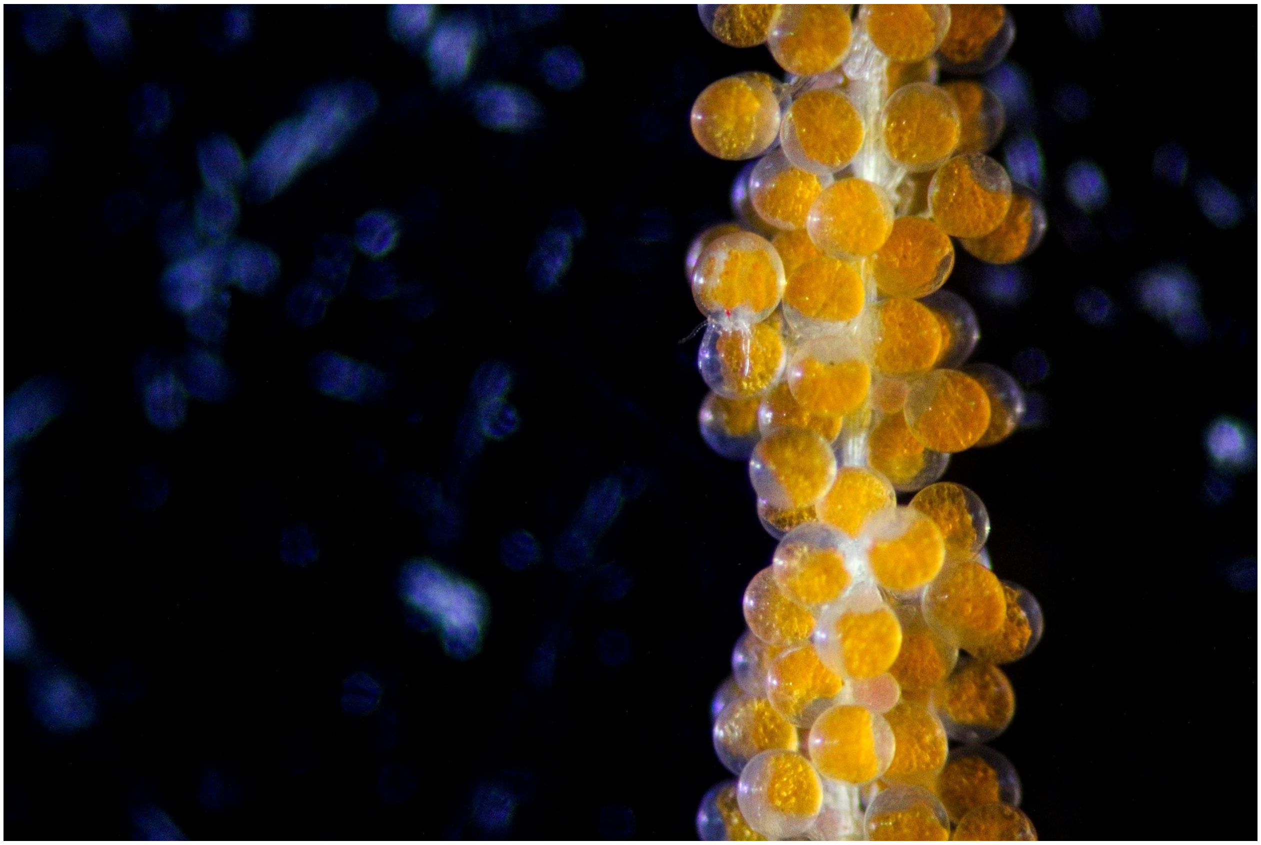

FIG S3. Adult nicothoid camouflaged amongst host crab eggs. Photo credits: J. E. Orli.

Section S4. Appendix References

Bloch, F., Gallien, L., 1933. Sur un Copépode parasite de la ponte de *Carcinus maenas* Pennant (*Lecithomyzon maenadis* n.g., n.sp.).

Connolly, C.J., 1929. A new copepod parasite *Choniosphaera cancrorum* gen. et sp. nov., representing a new genus, and its larval development. Proceedings of the Zoological Society of London.

Dang, Binh Thuy, S. Q. Tran, O. T. Truong, O. T. Kieu Le, and Q. D. H. Vu. 2022. "Species Diversity and Molecular Taxonomy of Symbiotic Crustaceans on *Portunus Pelagicu*s (Linnaeus, 1758) in Vietnam, with Remarks on Host Records and Morphological Variation." *Nauplius* 30: e2022027. https://doi.org/10.1590/2358-2936e2022027.

Fischer, W., 1956. Untersuchungen über einen für die deutsche Bucht neuen parasitären Copepoden: *Lecithomyzon maenadis* Bloch & Gallien (Familie Choniostomatidae) an *Carcinus maenas* Pennant (Crustacea Decapoda). Helgolander Wiss. Meeresunters 5, 326–352. https://doi.org/10.1007/BF01626175

Johnson, M.W., 1957. The Copepod *Choniosphaera cancrorum* Parasitizing a New Host, the Green Crab *Carcinides maenas*. The Journal of Parasitology 43, 470. https://doi.org/10.2307/3274678

Orli, J. O., S. Lecuona, G. Plewe, C. Gadler, A. M. Kuris, D. Tang, and Z. L. Zilz. 2025. Discovery of an unidentified species of nicothoid copepod infesting cancrid crabs in Santa Barbara, California. Zenodo. https://doi.org/10.5281/zenodo.10699477

Shields, J., Wood, R.-E., 1993. Impact of parasites on the reproduction and fecundity of the blue sand crab *Portunus pelagicus* from Moreton Bay, Australia. Mar. Ecol. Prog. Ser. 92, 159–170. https://doi.org/10.3354/meps092159

Shields, J.D., Okazaki, R.K., Kuris, A.M., 1990. Brood Mortality and Egg Predation by the Nemertean, *Carcinonemertes epialti*, on the Yellow Rock Crab, *Cancer anthonyi*, in Southern California. Can. J. Fish. Aquat. Sci. 47, 1275–1281.
